# One-shot genitalia are not an evolutionary dead end - Regained male polygamy in a sperm limited spider species

**DOI:** 10.1186/1471-2148-11-197

**Published:** 2011-07-08

**Authors:** Jutta M Schneider, Peter Michalik

**Affiliations:** 1 Biozentrum Grindel, Zoological Institute and Museum, University of Hamburg, Martin-Luther-King Platz 3, D-20146 Hamburg, Germany; 2 Department of General and Systematic Zoology, Zoological Institute and Museum, Ernst-Moritz-Arndt-University, J.-S.-Bach-Str. 11/12, D-17489 Greifswald, Germany

## Abstract

**Background:**

Monogynous mating systems with extremely low male mating rates have several independent evolutionary origins and are associated with drastic adaptations involving self-sacrifice, one-shot genitalia, genital damage, and termination of spermatogenesis immediately after maturation. The combination of such extreme traits likely restricts evolutionary potential perhaps up to the point of making low male mating rates irreversible and hence may constitute an evolutionary dead end. Here, we explore the case of a reversion to multiple mating from monogynous ancestry in golden orb-web spiders, *Nephila senegalensis*.

**Results:**

Male multiple mating is regained by the loss of genital damage and sexual cannibalism but spermatogenesis is terminated with maturation, restricting males to a single loading of their secondary mating organs and a fixed supply of sperm. However, males re-use their mating organs and by experimentally mating males to many females, we show that the sperm supply is divided between copulations without reloading the pedipalps.

**Conclusion:**

By portioning their precious sperm supply, males achieve an average mating rate of four females which effectively doubles the maximal mating rate of their ancestors. A heritage of one-shot genitalia does not completely restrict the potential to increase mating rates in *Nephila *although an upper limit is defined by the available sperm load. Future studies should now investigate how males use this potential in the field and identify selection pressures responsible for a reversal from monogynous to polygynous mating strategies.

## Background

In the absence of paternal care, the male sex is generally considered to show a larger variation in mating rates than the female sex [1,2]. Due to the small size of the male gametes, they can be produced at relatively low cost and in large numbers, and replenished after use. Classical sex roles do not apply in mating systems without paternal care in which males are nevertheless monogynous while females mate multiply [3]. In such cases, males are adapted to focus their entire mating effort on fertilising a single female. A monogynous mating strategy can invade if several conditions are fulfilled: firstly, a male biased sex ratio is required that reduces the average male paternity success to a value below one female. Secondly, an effective mechanism of paternity protection, associated with the monogynous strategy is essential to elevate the paternity success of a monogynist above the average [4]. Under certain conditions, monogyny can co-occur with bigyny, a strategy in which males mate with a maximum of two females [5].

Monogynous mating systems have several evolutionary origins across a wide range of taxonomic groups and seem to have evolved several times independently in spiders [3,6]. Several extreme adaptations including permanent attachment to the female in angler fish, damage to the genitals, e.g. in honey bees and spiders, as well as post-insemination sexual cannibalism co-evolved with monogyny [6]. Monogynous males have no residual reproductive value after they have mated with a single female and can invest maximally in any adaptation that increases their paternity. Associated phenomena, namely self-sacrifice and genital damage to the male, are best studied in spiders which are ideal model organisms for mating system research [7].

Male genitalia in spiders are paired secondary structures, the pedipalps, which the males charge with sperm after maturation [8]. The male releases sperm from the ventral genital pore onto a special silken structure, the sperm web. The pedipalps are dipped into the sperm-containing liquid and charged with the ejaculate. This process of sperm induction can be repeated between copulations when sperm contents in the pedipalps are depleted [8]. Based on the present state of knowledge most spider species re-charge their pedipalps - an ability which has been reduced in monogynous species of some families. Indeed, several species of the families Araneidae, Theriididae and Nephilidae have been found to terminate spermatogenesis before maturation resulting in a single sperm load per pedipalp without the option to recharge depleted pedipalps [9-11] - the trait was termed permanent sperm depletion (PSD) [11]. The pedipalps are charged only once, usually soon after maturation, and each pedipalp is generally used only once (one-shot genitalia). Since males of at least two of these species are strictly monogynous, terminal investment strategies evolved that enhance male fertilisation success with a single female. Accordingly, males of the monogynous species risk or even sacrifice their life to prolong copulation duration and they damage their pedipalps to protect their paternity against future rivals. In the theridiid species *Tidarren argo *the males even ectomise one of their two pedipalps during maturation and always die while attached to the female with the remaining pedipalp [12]. Hence, with the evolution of one-shot genitalia, the production of sperm after induction is no longer required and the costs of sperm production can be saved in favour of other body functions and demands [10]. In addition to self-sacrifice and genital damage, the termination of spermatogenesis at maturation may be another trait of monogamous spider males that limits their mating rates to one or two females [11]. The accumulation of extreme adaptations to monogyny may severely constrain the evolutionary potential to change back to higher mating rates and may thereby constitute an evolutionary dead end. To date reversals to a polygynous mating system have not been reported. However, there is at least anecdotal evidence of a differentiation in male mating rates within the spider genus *Nephila*.

In the family Nephilidae, genital damage and one-shot genitalia seem to have evolved relatively early and are a common pattern in the genera *Herennia *and *Nephilengys *[13]. In both genera males not only damage their sperm transferring structure, the embolic conductor, but they ectomise the entire pedipalp after copulation, an adaptation termed "eunuch phenomenon" [14,15]. Recent molecular and combined phylogenetic analyses suggest *Nephila *to be the sistergroup of *Nephilengys *+ *Herennia *and thus the "eunuch phenomenon" might be a synapomorphy for this clade [16,17] The genus *Nephila *contains 15 species and their relationships were addressed in recent phylogenetic studies which revealed that either *N. fenestrata *[18] or a clade with *N. pilipes *and *N. constricta *[17] is sister to all remaining *Nephila*. In any case, all three species show male genital damage [19]. Remaining *Nephila *species vary in whether male genitalia are damaged or not during copulation [18]. For example, the Australian species, *N. plumipes *is known for frequent genital damage while this does not occur in the sympatric *N. edulis *[20-22]. Likewise, the African *N. fenestrata *frequently breaks off the tip of the embolic conductor [23] while this has not been observed in the sympatric *N. senegalensis *(unpublished observations). The function of genital damage varies between species: while it serves as a mating plug in *N. fenestrata *[24] it does not prevent copulations and paternity of rival males in *N. plumipes *[21]. In at least two species, *N. inaurata *and *N. pilipes*, genital fragments of several males can be found in single genital openings of females [13,25]. The frequency of sexual cannibalism also varies between species and in several species males evolved tactics to survive female attacks. One such strategy is to mate opportunistically while the females are moulting or feeding. Opportunistic mating may be facultative as in *N. fenestrata *[26] and *N. clavipes *[27] but is obligate in *N. plumipes *and *N. inaurata *[21,25]. Furthermore, in several species that do not or rarely show genital damage, e.g. *N. clavipes, N. edulis, N. inaurata *and *N. senegalensis*, repeated use of pedipalps can be observed [27-29]. However, in *N. clavipes *males usually empty their entire sperm load in a single copulatory bout at least when copulating with virgin females and subsequent copulations occur but without sperm transfer [30]. Repeated use of the same pedipalp with the same female increases paternity share under sperm competition in *N. edulis *and in *N. senegalensis *(unpublished) but it is unknown whether this is achieved through the transfer of additional sperm or through other mechanisms that influence the paternity share such as the transfer of manipulative substances or copulatory courtship (Eberhard 1996).

Here we study the phenomenon of multiple insertions in male *N. senegalensis *from three angles. First, we investigate the morphology of the testes before and after maturation in order to test whether males of this species are indeed terminating spermatogenesis and thus are sperm limited or whether they may have regained the possibility to produce sperm as adults. Second, we ask whether males can fertilise eggs of several virgin females through the repeated use of their palps. Third, we assess whether females are polyandrous and whether receptivity is influenced by mating.

## Results

### (a) Male genital system

**Figure 1:**
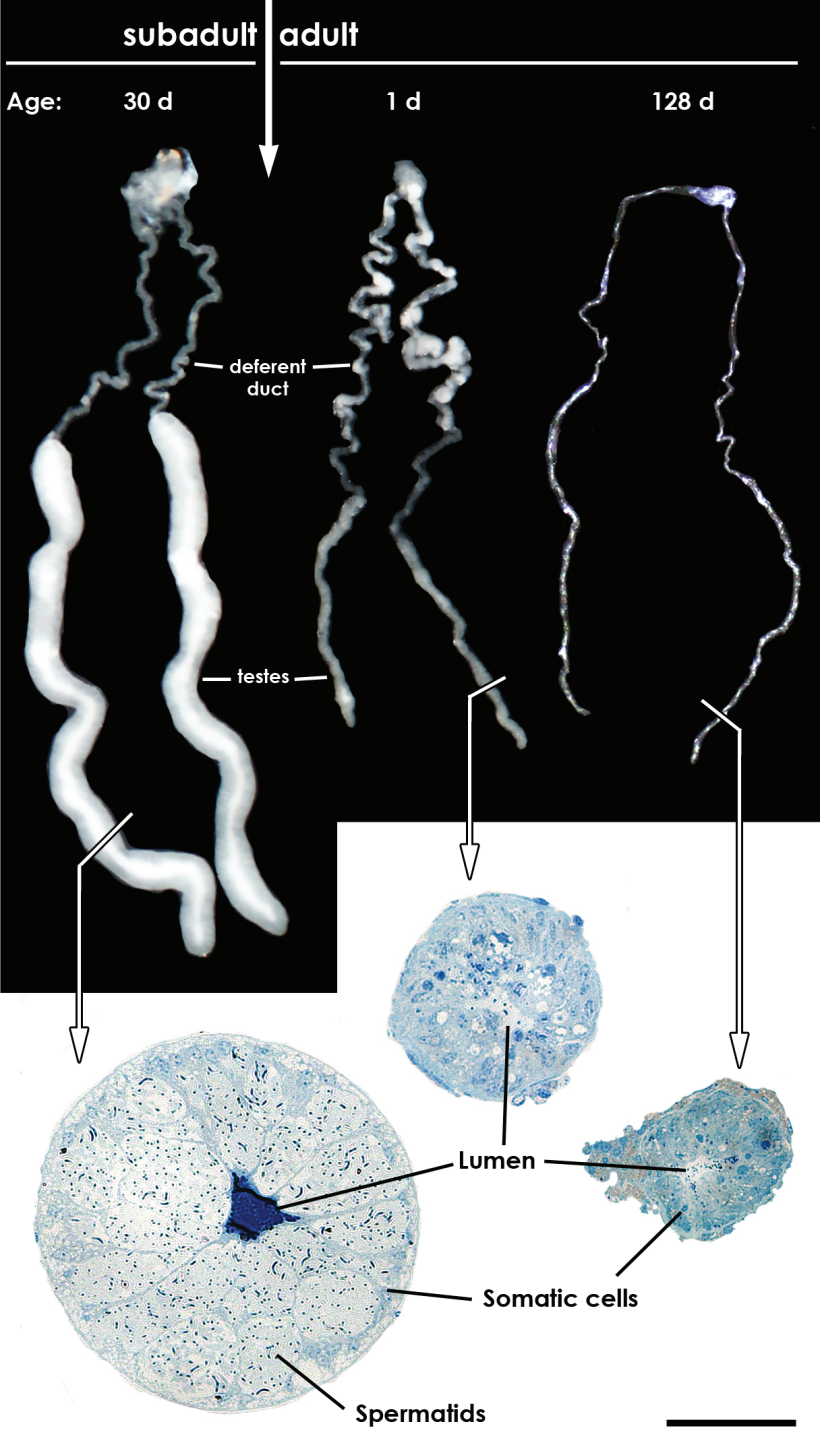
**Male genital system of *Nephila senegalensis *in subadult (30d after penultimate moult) and adult stage (1d and 128d after final moult)**. View of the whole genital system and stained cross-sections of the testis. As evident in the section of the testis of the subadult male the spermatids developing in cysts and the lumen is filled with dense secretion. In the adult testis only somatic tissue is present whereas generative tissue is completely absent. Scale bar: 50 μm.

As revealed by dissections and histological analyses, spermatogenesis is terminated before adulthood (Figure 1). Testis size decreased dramatically in adulthood and no generative tissue could be observed after final moult (as found in *N. clavipes*, [11]). Testes size keeps decreasing with days elapsed since the final moult and a regain of spermatogenesis was not observed (Figure 1). Spermatids, which develop in cysts surrounded by extensions of the somatic cells, are of the same developmental stage indicating a synchronous spermatogenesis (Figure 1).

### (b) Male polygamy

**Figure 2:**
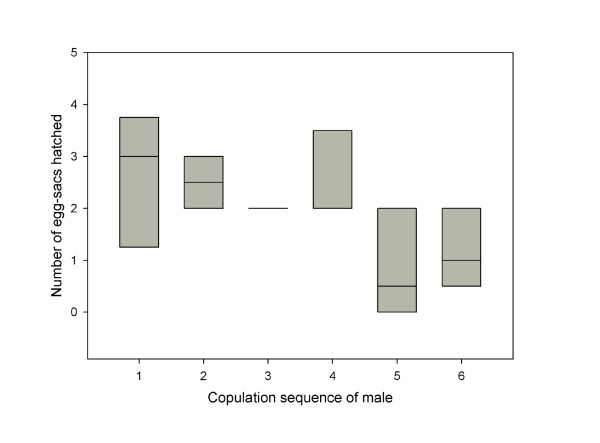
**Individual males were mated to 6 females in succession**. The number of fertilised egg-sacs that females produced after a single copulation is similar for the first 4 females and then declines. Box-Plots depict medians and interquartiles of 8 females per mating sequence.

Eight males were mated to a total of 55 females (minimum 5, maximum 9 females per male) and allowed to use one pedipalp once with every female. Reloading of pedipalps (sperm induction) was never observed between copulations. Females produced between 1 and 7 egg-sacs (most females produced 3 egg-sacs) before they died a natural death. Following a single copulation with a male, females produced on average 1.76 (SE = 0.16) fertilised egg-sacs. Fertilisation success decreased drastically after the 4^th ^trial of a male; while the first four mating partners of a male had similar fertilisation success, the egg-sacs of subsequent mating partners were less often fertilised (Figure 2). Individual males inseminated at least 3 and at most 5 females (mean = 4.6 ± 0.5) and fertilised between 8 and 16 egg-sacs (mean = 12.25 ± 1.0).

**Figure 3:**
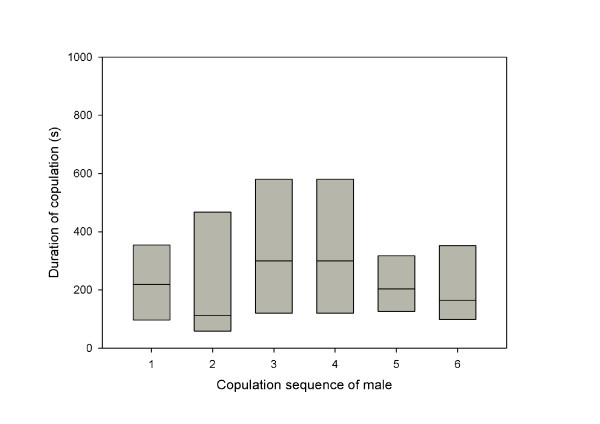
**Individual males were mated to 6 females in succession**. The duration of copulations varied (medians and interquartiles are shown, N = 8) but did not significantly differ between trials even though no or very little sperm was transferred to the 5^th ^and 6^th ^female of a male.

Copulation durations were not affected by the order of the mating trial (restricted to the first 6 trials due to sample sizes; male ID used as a random effect; ANOVA, F_5,39 _= 0.47, p = 0.79; Figure 3). This suggests that males show normal courtship and mating behaviour even if they are depleted of sperm.

Copulations were observed and the use of the two pedipalps was documented. Seven of 8 males used their left pedipalp first and 4 of these males re-used their left pedipalp with their 2^nd ^female. All males used both of their pedipalps; right pedipalps were used more than once by 7 of the 8 males, and left pedipalps were repeatedly used by 6 of 8 males. Right pedipalps were used 3.75 (SE = 0.67) times and left pedipalps 2.86 (SE = 0.52) times on average. The difference was not significant (Wilcoxon, Z = 0.96, p = 0.34).

The re-use of a pedipalp correlated significantly with the number of females that the males inseminated with this pedipalp (r^2 ^= 0.59, F_1,14 _= 20.49, p = 0.0005). With their right pedipalps males inseminated 2.38 females (SE = 0.50, range 1-5) and with their left pedipalp they inseminated two females (SE = 0.38, range 1-6). The difference was not significant (Wilcoxon, Z = 0.44, p = 0.66). In all cases in which a pedipalp was used more than once, the first two females inseminated with this palp produced fertilised clutches.

For each of the 8 males we compared the copulation of the pedipalp used during the first copulation. The duration of the first copulation varied around a mean of 277.1s (SE = 84.8) and was negatively although not significantly related to the frequency of re-use of the same pedipalp (r^2 ^= 0.38, F_1,6 _= 3.75, p = 0.1) and accordingly to the number of females fertilised with this pedipalp (r^2 ^= 0.44, F_1,6 _= 4.7, p = 0.07).

In a second experiment, we increased sperm limitation by amputating one pedipalp of 18 males. We investigated the re-use and the fertilisation potential of single pedipalps and exposed males to up to 4 females. From the results of the previous experiment we expected that male fertilise two females with a single pedipalp. Males generally used their pedipalp repeatedly although one male was cannibalised by his 2^nd ^female and another male could not be motivated to copulate with more than two females. The mean durations of copulations did not significantly differ between the four trials (ANOVA on trials 1 to 4, male ID as random effect: F_3,59 _= 2.47, p = 0.074).

For unknown reasons a large proportion of females in the 2^nd ^experiment suffered from complete or partial reproductive failure; several females died before oviposition and others produced egg-sacs that were empty or contained dried eggs. In the previous experiment, all males fertilised the eggs of the first female they mated with. In order to avoid errors by wrongly accounting reproductive failures to decisions of the males, we restricted our analysis of male fertilisation success to those males that inseminated at least the first female they copulated with. This reduced the sample size to 7 males and 28 copulations. The average number of females with fertilised egg-sacs per pedipalp was two (one male fertilised only 1 and 1 male 3 females) and no male succeeded to inseminate all 4 females. The use and fertilisation pattern of one-palped males matched the results of the previous experiment in which males were able to freely choose the pedipalps they used. The increased sperm limitation did not induce the males to change the patterns of sperm allocation. Both experiments suggest that most males split the sperm load in a single pedipalp into two portions.

If both experiments are combined, the repeated use of the single or firstly used pedipalp on fertilisation success can be analysed with a larger sample size. The duration of the first copulation significantly predicted whether 1, 2, 3 or 4 females were fertilised with the same pedipalp (multiple logistic model: χ^2^_2 _= 6.59, p = 0.037; copulation duration χ^2 ^= 5.91, p = 0.015; amputated yes/no: χ^2 ^= 0.11, p = 0.74). Males that inseminated a single female mated for a median of 442s (N = 3). Males that inseminated more than 2 females had a relatively shorter 1^st ^copulation (Median = 213s, N = 3) although not much different from cases in which sperm was divided between two females (median = 232s, N = 9).

### (c) Female polygamy

Several males were successively introduced to each female (range 6 - 13 males per female). All males were generally accepted as mating partners and there was no obvious decline in female receptivity. The number of males per female was only limited by the available individuals in our laboratory stock. An ANCOVA with female ID as random factor revealed that 37% of the variation in copulation duration was explained by the mean tibia-patella length of the males in that small males mated longer than large males (F_1,52 _= 5.78, p = 0.021) while neither the trial number (1-6) (F_5,57 _= 0.31, p = 0.9) nor the pedipalp used (F_1,52 _= 0.03, p = 0.86) were relevant. This suggests that female mating status (regardless of which of her paired spermathecae is considered) and the degree of polyandry do not prevent her from accepting copulations of normal length.

## Discussion

*Nephila senegalensis *males do not continue spermatogenesis in the adult stage and are unable to recharge their pedipalps between copulations (PSD [11]). This appears to be a heritage from their evolutionary history; the congener *N. clavipes *shows the same trait and so do species from the closely related genus *Nephilengys *[11]. The latter are strictly monogynous and possess one-shot genitalia [9]. These patterns suggest that PSD initially co-evolved with other adaptations to monogyny such as genital damage and one-shot genitalia [11].

In contrast to their ancestors who likely possessed one-shot genitalia [13,31,32], *N. senegalensis *males no longer damage their genitalia and are able to use their pedipalps repeatedly. Here we show that the repeated use of pedipalps indeed involves a portioning of the sperm supply within a single pedipalp. This is an important finding as this study also shows that males with empty pedipalps exhibit normal mating behaviour so that the occurrence of copulation cannot be equalised with sperm transfer. Through portioning of sperm, *N. senegalensis *more than doubled their maximal mating rates in comparison to their monogynous or bigynous relatives. Based on the current phylogenetic hypotheses [14,16-18], they have regained the potential for polygamy. It remains to be studied in nature whether males use this potential of multiple mating and move between females or whether they use repeated copulations to maximise their paternity success under sperm competition [29]. In the Australian *N. edulis *the dominant male will increase the frequency of copulation in a competitive situation [29]. A paternity study on *N. edulis *revealed that repeated copulations positively affected paternity success under sperm competition [22]. However, the paternity share was not directly related to the repeated use of a pedipalp but to the total copulation duration, irrespective in how many copulatory bouts took place [22]. Interestingly, repeated copulations were used by large males while small males copulated for longer [33]. However, if two males of similar size competed, the frequency of copulation was highest [33]. The size and competition dependent alternative tactics are proposed to maintain the size variation in male *Nephila *[33-35]. Context dependent mating strategies are also likely in *N. senegalensis *and may influence sperm allocation as well.

In most cases, males allocated the sperm load of one palp into two portions although more or less portions are possible. A congener, *N. clavipes*, also known to reuse pedipalps despite sperm limitation [11,27], deplete their sperm supply with virgin females [30] while this occurred very rarely in *N. senegalensis*. Indeed, the length of the first copulation determined how many portions are left for future copulations with the same pedipalp. Here we suggest that *N. senegalensis *show a prudent use of their sperm supply during their first copulation and always retain a quantity of their limited sperm supply for future copulations.

Males readily mated with several females but not every copulation resulted in fertilisation success. Especially twice mated males appeared to copulate for a normal length without transferring any sperm. Likewise, females copulated with sperm depleted males even though they did not benefit at all. In our laboratory setting, females were highly polyandrous and accepted numerous males for copulations. One possible benefit of polyandry may be that females secure a sufficient sperm supply. Multiple mating will be adaptive if males are mobile and multiply mate in nature and if females have no mechanism to detect whether males transfer any sperm or not. It is unknown how relevant this is under natural conditions and extensive field studies are required to answer this.

In nature, males accumulate on the large webs of *Nephila *females and compete for copulations. This is a general pattern in the genus and is considered one of the most important requirements for the evolution of monogyny [4,6]. However, a monogynous strategy can only invade and be stable, if it is associated with a potent mechanism of paternity protection [4]. A common trait in this respect that evolved independently in several spider families is the application of a mating plug through damaging the genitalia [19].

*N. fenestrata *is the only species in which males still use genitalia fragments to close the female genital opening. In no other *Nephila *species studied to date genital damage leads to the application of a mating plug that is effective in excluding sperm competition. Provided the high incidence of competition, species with one-shot genitalia but without the plug function are expected to evolve alternative measures of paternity protection. Mate guarding or the transfer of accessory substances that manipulate female receptivity are such strategies as shown for several insect species [36,37]. Species without genital damage have the additional option to inseminate several females but if a male depletes the sperm in a pedipalp during a single copulation this will restrict males to the maximum of two different mates, one with each pedipalp. *N. senegalensis *overcomes this limitation at least to some degree by portioning the sperm in each pedipalp. To date it is unknown which selection pressures are responsible for the loss of genital damage and the recovery of a potential for polygamy. A reduction in the effectiveness of genital fragments as mating plugs is explained by sexual conflict. Female and male genital morphology co-evolved in that female genital openings could no longer be plugged because male embolic conductors became thinner and bypass each other while female genital openings became wider [14].

## Conclusion

The case of the Nephilidae remains to be among the most promising systems to explore the role of antagonistic co-evolution in concert with extreme male mating strategies. Current knowledge suggests that despite an evolutionary heritage of behavioural, morphological and physiological adaptations to monogyny, males can reverse to polygamy by overcoming sexual cannibalism and genital damage and while they did not revert to adult sperm production, they developed the potential to an economical use of their limited sperm supply.

## Methods

Spiders were F_1 _and F_2 _offspring derived from wild-caught individuals collected in 2008 near Craddock, Eastern Cape Province, South Africa. Juvenile spiders were kept in individual plastic cups (250 ml) and while males remained in cups, subadult and adult females were housed in separate Perspex frames (60 cm × 60 cm × 12 cm), where they built typical orb-webs. Females were sprayed with water on 5 days per week, fed 5-8 *Calliphora *sp. flies 2-3 times per week, and weighed on the day after their final moult. After mating, females were transferred to individual plastic cups (400 ml), where they laid eggs. After death of a female, we used callipers to take the tibia-patella length of a foreleg as a measure of its fixed body size. Males were maintained in individual cups (250 ml) on a diet of *Drosophila*. Males were fed *ad libitum *twice per week and sprayed with water on 5 days per week. On the day after the final moult, each male was weighed and after the male had died the tibia-patella length of both forelegs was measured under a dissecting microscope using the measuring tool of Leica IM500. Measurements were taken of all males involved in the below experiments. Egg-sacs were removed from cups and incubated in individual containers at room temperature for 5 weeks and then placed in 70% ethanol. Egg-sacs were carefully opened and inspected for the presence of hatchlings. Hatchlings will stay in the egg-sac for a few days after they hatched from the eggs. Eggs that have not developed by that time will not hatch. If hatchlings were found in an estimated proportion above 50%, the egg-sac was considered to be fertilised (most egg-sacs had above 80% hatching rate).

### (a) Morphology of the male genital system

Subadult (5, 30 days after penultimate moult) and virgin adult (1, 2, 3, 4, 5, 28, 128, 147 days after final moult) male specimens were dissected in phosphate buffer (0.1 M, pH 7.2) with 1.8% sucrose added (PB). The isolated genital systems were fixed in 2.5% glutaraldehyde (Merck Chemicals Ltd., Nottingham, UK) in PB and documented using Leica EZ 4D stereomicroscope or Olympus ZX 7 stereomicroscope with an Olympus DP 10 digital camera. Afterwards, the samples were post-fixed in PB buffered 2% OsO_4 _(SERVA Electrophoresis GmbH, Heidelberg, Germany), washed in PB, dehydrated in graded ethanol and embedded in Spurr's resin [38]. Semi-thin sections (700 nm) were made with a Diatome HistoJumbo diamond knife at a Leica ultramicrotome UCT and stained according to Richardson et al. [39]. Sections were documented using a Zeiss Mcr digital camera mounted on an Olympus BX60 compound microscope. For more details see [11]. Vouchers are deposited in the Zoological Institute and Museum Greifswald, Germany (ZIMG).

### (b) Male polygamy

Virgin males were assigned to mate with several virgin females in succession. Females always received a fly before the male was carefully introduced in one of the upper corners of the web. Thereby the risk of losing males due to sexual cannibalism is minimized [26]. Courtship and copulation were closely observed and a single copulation was permitted per female. We noted which pedipalp the male used and measured the duration of the copulation with a stop watch. Copulation ended by the male walking off the female or by the female brushing the male off. After the end of the first copulation, the male was carefully removed from the web with a paint-brush and returned to his plastic cup. Sometimes on the same day but otherwise on the following days, the male was placed onto the web of another virgin female. The procedure was repeated until the male died a natural death (one male fell victim to sexual cannibalism), until the male did not show any courtship for a series of at least two trials, or until we ran out of virgin females.

**In a first experiment**, we used virgin males and mated them to a minimum of 5 and a maximum of 9 females. In this treatment, males were free in the choice of which of their two pedipalps they used. Copulations were observed and the used pedipalp was documented.

**In a second experiment**, we tested how many females males fertilize if they only have a single pedipalp, hence have an even stronger limitation of sperm. After a preset schedule, we amputated either the left or the right pedipalp of a virgin male. Amputation was induced by squeezing the male pedipalp with forceps until the male ectomised the appendage. In this way, haemolymph leakage is minimized or even absent. The method is well established in our laboratory and is not known to influence behaviour and survival of males. The amputated males (half eunuchs) were mated with 5 females in succession.

### (c) Female polygamy

Nine females were mated with several males in succession (6 to 13) and each males was allowed a single copulation with one pedipalp. Courtship and copulation duration were documented as well as the pedipalp each male used. Males and females were otherwise treated in the same way as described above.

### (d) Statistical Analysis

Data were analyzed with JMP 7. Sample sizes can vary due to missing data or exclusions of data. Some analyses that involve comparisons of repeated trials are restricted to the first 6 repeats because only few individuals were mated more than 6 times. Exclusions are generally made explicit with the results. In one-way tests, trial number was defined as an ordinal variable. Data on the duration of copulation were log-transformed to achieve a normal distribution. All continuous data sets used for parametric statistics are normally distributed and have homogeneous variances.

## Competing interests

The authors declare that they have no competing interests.

## Authors' contributions

JMS conceived the experiments, analyed the data and wrote the paper. PM conducted the histology and contributed to writing the text. All authors have read and approved the final manuscript.
